# Proteomic profiling of longitudinal changes in kidney function among middle-aged and older men and women: the KORA S4/F4/FF4 study

**DOI:** 10.1186/s12916-023-02962-z

**Published:** 2023-07-05

**Authors:** Jie-sheng Lin, Jana Nano, Agnese Petrera, Stefanie M. Hauck, Tanja Zeller, Wolfgang Koenig, Christian L. Müller, Annette Peters, Barbara Thorand

**Affiliations:** 1 Institute of Epidemiology, Helmholtz Zentrum München, German Research Center for Environmental Health (GmbH), Ingolstädter Landstraße 1, 85764 Neuherberg, Germany; 2 Institute for Medical Information Processing, Biometry, and Epidemiology (IBE), Faculty of Medicine, LMU Munich, Pettenkofer School of Public Health, Munich, Germany; 3 Chair of Epidemiology, Institute for Medical Information Processing, Biometry, and Epidemiology (IBE), Ludwig-Maximilians-Universität München, Munich, Germany; 4 Metabolomics and Proteomics Core, Helmholtz Zentrum München, German Research Center for Environmental Health (GmbH), Neuherberg, Germany; 5 University Center of Cardiovascular Science, University Heart and Vascular Center, University Medical Center Hamburg-Eppendorf, Hamburg, Germany; 6 German Center for Cardiovascular Research (DZHK), Partner Site Hamburg, Hamburg, Germany; 7 Deutsches Herzzentrum München, Technische Universität München, Munich, Germany; 8 German Center for Cardiovascular Research (DZHK), Partner Site Munich Heart Alliance, Munich, Germany; 9 Institute of Epidemiology and Medical Biometry, University of Ulm, Ulm, Germany; 10 Institute of Computational Biology, Helmholtz Zentrum München, German Research Center for Environmental Health (GmbH), Neuherberg, Germany; 11 Helmholtz AI, Helmholtz Zentrum München, German Research Center for Environmental Health (GmbH), Neuherberg, Germany; 12 Department of Statistics, Ludwig-Maximilians-Universität München, Munich, Germany; 13 Center for Computational Mathematics, Flatiron Institute, New York, USA; 14 German Center for Diabetes Research (DZD), Partner München-Neuherberg, Neuherberg, Germany

**Keywords:** Proteomics, Glomerular filtration rate, Chronic kidney disease, Cohort study, Mendelian randomization

## Abstract

**Background:**

Due to the asymptomatic nature of the early stages, chronic kidney disease (CKD) is usually diagnosed at late stages and lacks targeted therapy, highlighting the need for new biomarkers to better understand its pathophysiology and to be used for early diagnosis and therapeutic targets. Given the close relationship between CKD and cardiovascular disease (CVD), we investigated the associations of 233 CVD- and inflammation-related plasma proteins with kidney function decline and aimed to assess whether the observed associations are causal.

**Methods:**

We included 1140 participants, aged 55–74 years at baseline, from the Cooperative Health Research in the Region of Augsburg (KORA) cohort study, with a median follow-up time of 13.4 years and 2 follow-up visits. We measured 233 plasma proteins using a proximity extension assay at baseline. In the discovery analysis, linear regression models were used to estimate the associations of 233 proteins with the annual rate of change in creatinine-based estimated glomerular filtration rate (eGFRcr). We further investigated the association of eGFRcr-associated proteins with the annual rate of change in cystatin C-based eGFR (eGFRcys) and eGFRcr-based incident CKD. Two-sample Mendelian randomization was used to infer causality.

**Results:**

In the fully adjusted model, 66 out of 233 proteins were inversely associated with the annual rate of change in eGFRcr, indicating that higher baseline protein levels were associated with faster eGFRcr decline. Among these 66 proteins, 21 proteins were associated with both the annual rate of change in eGFRcys and incident CKD. Mendelian randomization analyses on these 21 proteins suggest a potential causal association of higher tumor necrosis factor receptor superfamily member 11A (TNFRSF11A) level with eGFR decline.

**Conclusions:**

We reported 21 proteins associated with kidney function decline and incident CKD and provided preliminary evidence suggesting a potential causal association between TNFRSF11A and kidney function decline. Further Mendelian randomization studies are needed to establish a conclusive causal association.

**Supplementary Information:**

The online version contains supplementary material available at 10.1186/s12916-023-02962-z.

## Background

Chronic kidney disease (CKD), which is characterized by a progressive loss in kidney function over months or years [1], affected approximately 9.1% of the general population globally in 2017 [2]. Estimated glomerular filtration rate (eGFR), together with albuminuria and blood urea nitrogen, are the most commonly used indicators to evaluate kidney function and diagnose CKD in clinical practice. Kidney function declines with aging, while early stages of CKD remain asymptomatic, resulting in CKD usually being diagnosed in late stages. Besides, there is no targeted therapy for CKD beyond the management of its risk factors, such as diabetes and hypertension. The pathophysiology leading to CKD is not completely understood, and thus, there is a pressing need to identify new biomarkers that may provide new insight into the underlying pathophysiology of CKD, help in early diagnosis, and potentially be used as therapeutic targets.

It has been well documented that CKD is strongly related to cardiovascular diseases (CVD), and they share common mechanisms such as oxidative stress and inflammation [3, 4]. Thus, targeting CVD- and inflammation-related biomarkers may provide a valuable opportunity to identify biomarkers likely to be involved in the pathophysiology of CKD. Advances in proteomics technology, such as proximity extension assay technology using the Olink platform [5], make it possible to measure a large number of targeted biomarkers simultaneously.

An increasingly large number of studies investigating the associations of urinary and circulating proteomic biomarkers with kidney function and the progression of kidney disease have been published in the past decade [6–16]. We have previously identified and replicated 42 proteins associated with kidney function from a panel of inflammatory proteins, and revealed several pathophysiological pathways related to kidney disease using pathway analysis, highlighting the importance of investigating proteomics profiling in the general population [15]. A longitudinal study applying a large proteomics approach also identified novel biomarkers of progression to kidney failure in diabetic patients [16].

The present study aimed to investigate the associations of 233 CVD- and inflammation-related plasma proteins with longitudinal changes in kidney function in a community-based prospective cohort of middle-aged and older adults to uncover biomarkers and pathways involved in longitudinal kidney function decline and CKD development. Furthermore, we aimed to investigate whether the observed associations were potentially causal by using a two-sample Mendelian randomization (MR) design.

## Methods

### Study population

**Fig. 1:**
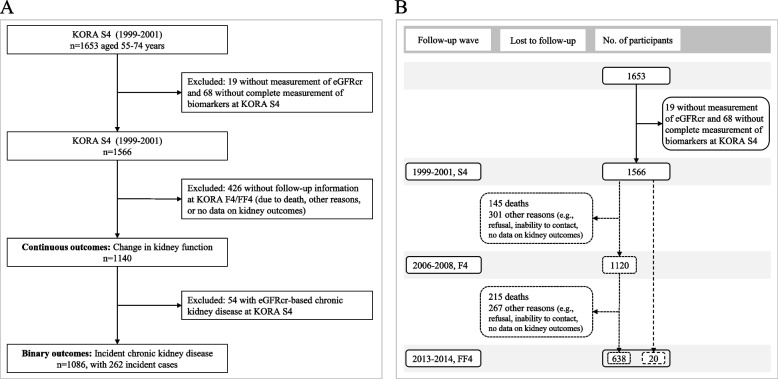
Flowchart of study participants. Abbreviations: eGFRcr, creatinine-based estimated glomerular filtration rate; KORA S4/F4/FF4, the Cooperative Health Research in the Region of Augsburg (KORA) cohort study at baseline ( S4), first follow-up (F4) and second follow-up (FF4)

This study was based on the Cooperative Health Research in the Region of Augsburg (KORA) S4/F4/FF4 cohort study [17, 18]. A total of 4261 adults, aged 25–74 years, were included at baseline between 1999 and 2001 (S4) in Germany. Follow-up examinations were conducted after 7 years (F4) and after 14 years (FF4). The present analysis was restricted to participants aged 55–74 years at S4 (*N* = 1653). The flow chart of study participants is presented in Fig. 1. Participants without data on creatinine-based estimated glomerular filtration rate (eGFRcr, *n* = 19) or complete proteomics measurements (*n* = 68) at KORA S4 were excluded, leaving a total of 1566 participants at baseline. For the longitudinal analysis, participants without follow-up information on eGFRcr at both F4 and FF4 (*n* = 426) were further excluded. Finally, a total of 1140 participants were included, with a median follow-up time of 13.4 (25^th^ percentile: 7.1, 75^th^ percentile: 13.5) years (Fig. 1A). Among these 1140 participants, 638 participants were both followed up at F4 and FF4, 482 participants were only followed up at F4, and 20 participants were only followed up at FF4 (Fig. 1B). Participants with eGFRcr-based CKD at baseline (*n* = 54) were further excluded when investigating the associations of proteomic biomarkers with incident CKD. The KORA S4, F4, and FF4 studies were approved by the local ethical committee (Number: 99186) and all participants gave written informed consent.

### Measurement of proteomic biomarkers

Plasma samples of baseline participants aged 55–74 years were used to measure proteomic biomarkers. Three Olink panels, including the Proseek Multiplex CVD II, CVD III, and Inflammation panels (Olink, Upsala, Sweden), each covering 92 proteins, were measured using proximity extension assay technology [5]. The assay allows the relative quantification of analyte concentrations and was given as normalized protein expression values on a log2 scale, with higher expression values corresponding to higher protein levels. Details of the measurement process and exclusion of the proteins (e.g., proteins with more than 25% of all data below the limit of detection were excluded) have been reported elsewhere [19, 20]. A total of 233 proteins were included in this study (Additional file 1: Table S1). The Z-score transformation was conducted for all values of proteins in 1566 participants with complete proteomics measurements at baseline, which allows comparing the magnitude of the effect sizes across proteins and Z-score transformation was appropriate for most of our proteins after evaluating the distribution of each protein.

### Measurement of kidney function and CKD

The primary outcomes in the present study were the annual rate of change in eGFRcr and incident eGFRcr-based CKD, given their availability at KORA S4, F4, and FF4. Creatinine was assessed in fresh serum using an enzymatic method at S4 (CREA plus, Boehringer, Mannheim, Germany), a modified kinetic rate Jaffe method at F4 and the first part of FF4 (CREA Flex, Dade Behring / Siemens Healthcare Diagnostics Products GmbH), and a Jaffe method for the second part of FF4 (Cobas 8000 instrument, Roche Diagnostics, Mannheim, Germany). Serum creatinine at KORA F4 and FF4 (part 1) were Isotope-Dilution Mass Spectrometry standardized. Kidney function was primarily assessed by eGFRcr calculated using the Chronic Kidney Disease Epidemiology Collaboration (CKD-EPI) Eq. 2009 [21]. A CKD case was defined as eGFRcr < 60 ml/min per 1.73m^2^ [1]. Participants free of CKD at S4 who had CKD at F4 or FF4 were defined as incident cases. In addition, the new race-free CKD-EPI Eq. 2021 [22] was used to calculate eGFRcr for supplementary analyses. Cystatin C-based eGFR (eGFRcys) was calculated using CKD-EPI cystatin C Eq. 2012 [23] and used for confirmation of significant associations. In comparison to eGFRcr, there were 278, 5, and 17 missing values on eGFRcys at S4, F4, and FF4, respectively, and imputation of missing values of eGFRcys was conducted by using a linear mixed-effects model. Additionally, urine albumin to creatinine ratio (UACR) was also available at F4. Details on measurements of UACR and cystatin C and imputation of missing values of eGFRcys are presented in Additional file 2: Text S1.

### Covariates

In all surveys, standardized face-to-face interviews were conducted by trained staff [17], gathering the following information: age, sex, physical activity (active/inactive), smoking status (never/former/current smoker), alcohol consumption (0/0–20.0/ >  = 20 g/d), use of antihypertensive medication (yes/no), use of lipid-lowering medication (yes/no), prevalent diabetes (yes/no), prevalent cardiovascular diseases (yes/no), and fasting status (fasting for 8 h or more before blood was taken, yes/no) [24, 25]. Participants who were non-fasting before blood was taken were predominantly participants with diabetes. Anthropometric indices and blood pressure were measured based on standard protocols. High-density lipoprotein-cholesterol and triglycerides were measured in serum on Hitachi 717/917 (Boehringer, Mannheim, Germany), respectively.

### Statistical analysis

Continuous variables were reported as mean (standard deviation, SD) for normally distributed data and median (interquartile range) for skewed data. Categorical variables were presented as total numbers with the corresponding percentage.

### Identification and confirmation of change in eGFR-associated proteins

For participants with eGFR measured at more than one visit (*n* = 1140), the annual rate of change in eGFR was calculated as β coefficients from linear regression of eGFR regressed against age at the time of eGFR measurement for each participant, and thus, each participant had 1 annual rate of change in eGFR (Additional file 2: Figure S1). Additional file 2: Figure S2 shows the flowchart of statistical analyses. In the discovery analysis, linear regression models were used to estimate the associations of the 233 proteins with the annual rate of change in eGFRcr. Two models were constructed: model 1 adjusted for age, sex, and eGFRcr at baseline; model 2 further adjusted for body mass index, physical activity, smoking status, alcohol consumption, systolic blood pressure, use of antihypertensive medication, triglycerides (naturally log-transformed), high-density lipoprotein cholesterol, use of lipid-lowering medication, prevalent diabetes, prevalent cardiovascular diseases, and fasting status at baseline. The 66 biomarkers significantly associated with the annual rate of change in eGFRcr were taken to investigate their associations with the annual rate of change in eGFRcys. Given the high proportion of missing values at FF4, we did not use linear mixed-effect models. Benjamini–Hochberg false-discovery rate (FDR) was performed for multiple testing correction, and FDR < 0.05 was considered statistically significant [26]. FDR seemed to be more appropriate to correct for multiple testing in our large-scale proteomics exploratory study, because it controls the rate of false positives while still allowing for the identification of a number of potential biomarkers.

Several sensitivity analyses of associations between the 66 significant biomarkers and the annual rate of change in eGFRcr were conducted based on model 2 described above. The analyses were repeated after exclusion of participants who were non-fasting at the time of blood sampling (*n* = 113), exclusion of participants who had CKD at baseline (*n* = 54), exclusion of participants who had an increase in eGFRcr during follow-up (*n* = 151), or further adjusted for UACR at F4 to control for the confounding effect of albuminuria (UACR values were unavailable at S4). To partially address bias caused by loss to follow-up (due to death or other reasons, Fig. 1B), the inverse probability weighting method [27] was used to calculate a weight for each participant. Each participant’s probability of loss to follow-up (P1) was estimated by logistic regression with loss to follow-up (yes/no) as outcomes, including baseline covariates in the above model 2 as predictors. Inverse probability weighting-weight was calculated as 1/(1-P1) and these weights were applied in model 2 (details are presented in Additional file 2: Text S2).

To further assess the robustness of our results on the annual rate of change in eGFRcr, rapid decline in eGFRcr (yes/no), which was defined as the annual rate of change in eGFRcr < -3 ml/min/1.73 m^2^/year [28], was used to investigate associations with the 66 eGFRcr-associated biomarkers in logistic regressions, adjusting for the same covariates as in the above model 2.

### Identification of incident CKD-associated proteins

The 66 proteins significantly associated with the annual rate of change in eGFRcr were taken to investigate their associations with eGFRcr-based incident CKD, using interval-censored Cox regression models (500 bootstrap samples were used to construct 95% confidence intervals), adjusted for the same covariates in the above model 2, using R package “icenReg v.2.0.15” [29]. Cox proportional hazards models were not appropriate, because we did not know the exact time point of CKD occurrence. In sensitivity analyses, incident eGFRcr-based CKD cases were redefined as follows: Participants free of CKD at S4 or F4, respectively, had to have more than 25% decline in eGFRcr together with eGFRcr < 60 ml/min per 1.73m^2^ at the following follow-up (i.e., F4 or FF4), or participants free of CKD at S4 had to have more than 50% decline in eGFRcr together with eGFRcr < 60 ml/min per 1.73m^2^ at FF4 [28]. The controls were defined as eGFRcr ≥ 60 ml/min per 1.73m^2^ at S4, F4, and FF4.

The proteins consistently associated with the annual rate of change in eGFRcr, the annual rate of change in eGFRcys, and incident CKD were further investigated regarding their associations with UACR at F4 using linear regression. To annotate druggable targets of the identified proteins, related information (e.g., known drugs, corresponding diseases or indications, and clinical trials status) was gathered from Open Targets Platform (https://platform.opentargets.org/) [30] based on their UniProt_IDs. In order to examine the novelty of the identified proteins, relevant publications were searched to check whether the identified proteins had been previously reported to be associated with kidney function and/or CKD.

### Mendelian randomization analysis

We applied a two-sample MR design using the largest genome-wide association studies (GWAS) results to date. Additional file 2: Figure S3 shows the process of MR analysis, and details of MR analysis are described in Additional file 2: Text S3 [31–37]. Briefly, selection of single nucleotide polymorphisms (SNPs) for proteins [31] and extraction of SNPs-eGFR decline associations [32] were from European ancestry population-based GWAS. To test the assumption of MR that instrumental variables are not associated with confounders, associations between selected SNPs and other traits were searched for in the PhenoScanner V2 [33]. One SNP (rs198389) was excluded given its associations with blood pressure (Additional file 1: Table S2), leaving 17 proteins for MR analysis (Additional file 1: Table S3). Wald ratio was calculated since only one SNP was available for each protein. MR analyses were performed using R package “TwoSampleMR v.0.5.6” [34].

### Pathway enrichment analysis

To characterize biological pathways that are enriched for the identified proteins, a Gene Ontology enrichment analysis was performed, using R package “clusterProfiler v.4.0.5” [38]. To investigate the potential biological pathways linking identified biomarkers and kidney function, the analysis was limited to the biological process subontology, using Fisher's exact test.

All analyses were conducted by R version 4.1.0 (R Development Core Team, Vienna, Austria) and RStudio version 1.4.1717 (RStudio, Boston, MA, USA).

## Results

### Characteristics of the study population

Table 1 shows the baseline characteristics of participants. The 1140 participants had a mean age of 63.3 (SD, 5.36) years. The median annual rate of change in eGFRcr was -1.04 ml/min/1.73 m^2^/year (Additional file 2: Figure S4A). Characteristics and kidney function of all participants over the study period are shown in Additional file 1: Table S4. The mean eGFRcr was 82.9 (SD, 12.2) ml/min/1.73 m^2^ at S4 and declined to 67.6 (SD, 15.3) ml/min/1.73 m^2^ at FF4. Figure 1B shows the number of participants and reasons for loss to follow-up and Additional file 1: Table S5 shows baseline characteristics for participants with and without follow-up information on eGFRcr. Participants lost to follow-up tended to be older, were more frequently smokers, consumed less alcohol, were less physically active, and had higher systolic blood pressure and prevalence of diabetes.

**Table 1:** Baseline characteristics of participants

	**Total (*N* = 1140)**	**Non-CKD cases** **(*N* = 824)**^**b**^	**Future CKD cases** **(*N* = 262)**^**b**^
	**Mean (standard deviation) or number (%)**		
Age (years)	63.3 (5.36)	62.3 (5.17)	65.5 (5.01)
Sex, N(%) female	556 (48.8)	400 (48.5)	125 (47.7)
Body mass index (kg/m^2^)	28.4 (4.25)	28.2 (4.22)	28.7 (4.45)
Smoking status, N (%)			
Never smoker	560 (49.1)	304 (36.9)	112 (42.7)
Former smoker	440 (38.6)	415 (50.4)	118 (45.1)
Current smoker	140 (12.3)	105 (12.7)	32 (12.2)
Alcohol consumption			
No alcohol consumption	291 (25.5)	194 (23.5)	76 (29.0)
> 0 and < 20 g/day	462 (40.5)	343 (41.7)	98 (37.4)
≥ 20 g/day	387 (33.9)	287 (34.8)	88 (33.6)
Physically active, N (%)	509 (44.6)	362 (43.9)	126 (48.1)
Systolic blood pressure (mmHg)	135.1 (19.8)	133.8 (19.3)	138.5 (21.3)
Use of antihypertensive medication, N (%)	388 (34.0)	228 (27.7)	126 (48.1)
Hypertension, N (%)	615 (53.9)	405 (49.2)	175 (66.8)
Triglycerides (mmol/L) ^a^	1.35 (0.93)	1.31 (0.97)	1.35 (0.90)
High-density lipoprotein cholesterol (mmol/L)	1.50 (0.43)	1.52 (0.43)	1.45 (0.42)
Use of lipid-lowering medication, N (%)	127 (11.1)	84 (10.2)	31 (11.8)
Type 2 diabetes, N (%)	94 (8.20)	53 (6.40)	32 (12.2)
Cardiovascular diseases, N (%)	135 (11.8)	84 (10.2)	37 (14.1)
Fasting status, N (%)	1027 (90.1)	760 (92.2)	221 (84.4)
eGFRcr (ml/min/1.73 m^2^)	82.9 (12.2)	86.6 (9.83)	77.1 (9.87)
eGFRcys (ml/min/1.73 m^2^)	81.1 (15.2)	84.9 (13.7)	73.6 (13.2)
Annual rate of change in eGFRcr (ml/min/1.73 m^2^/year) ^a^	-1.04 (1.35)	-0.86 (1.06)	-2.05 (1.36)
Annual rate of change in eGFRcys (ml/min/1.73 m^2^/year) ^a^	-1.13 (1.47)	-0.93 (1.44)	-1.78 (1.40)

*Abbreviations***:** *CKD* Chronic kidney disease, *eGFRcr* Creatinine-based estimated glomerular filtration rate; *eGFRcys* Cystatin C-based estimated glomerular filtration rate

^a^ Reported as median (interquartile range)

^b^ Participants with eGFRcr-based CKD at baseline (*n* = 54) were excluded

### Associations of proteomic biomarkers with kidney function decline

**Fig. 2:**
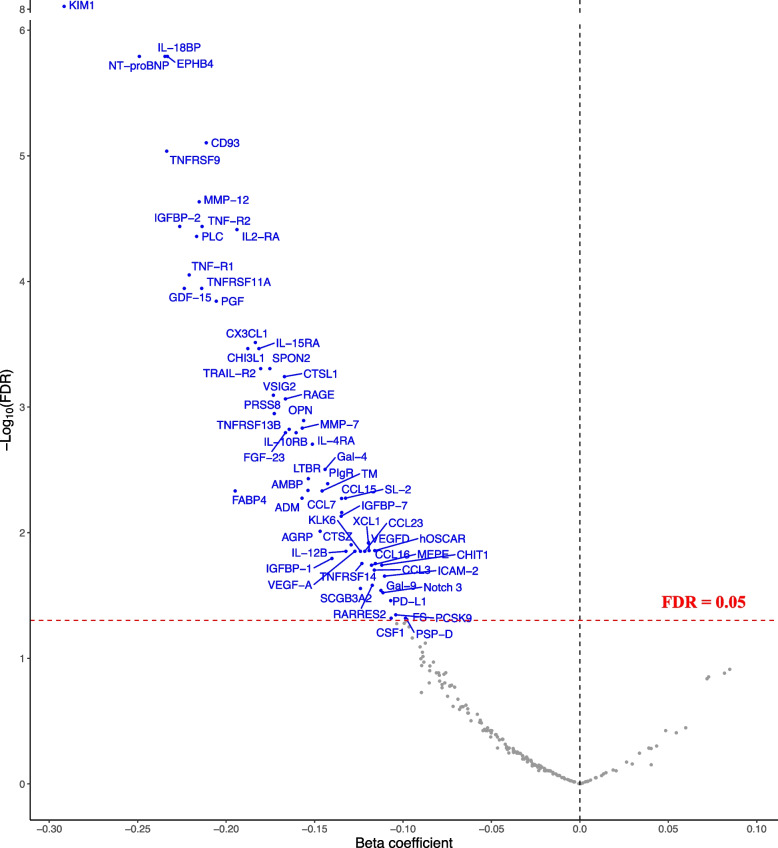
Association of 233 proteomic biomarkers with the annual rate of change in eGFRcr. Detailed results of beta coefficients and FDR for the association of 233 proteins with change in eGFRcr are presented in Additional file 1: Table S6 for model 2. Abbreviations: eGFRcr, creatinine-based estimated glomerular filtration rate; FDR, Benjamini–Hochberg false-discovery rate. Full names of the biomarkers can be found in Additional file 1: Table S1

After adjustment for age, sex, and baseline eGFRcr in model 1, 95 out of 233 biomarkers were inversely associated with the annual rate of change in eGFRcr (FDR < 0.05), which means that in comparison to participants with lower levels of biomarkers at baseline, participants with higher levels of biomarkers had a faster decline in eGFRcr (Additional file 1: Table S6). After adjustment for additional covariates in model 2, 66 biomarkers showed inverse associations with change in eGFRcr (62 of these were also significant in model 1) (Additional file 1: Table S6 & Fig. 2). The top 3 biomarkers with the lowest FDR were KIM1 (FDR = 9.51E-09, β = -0.292), NT-proBNP (FDR = 1.62E-06, β = -0.249), and EPHB4 (FDR = 1.62E-06, β = -0.233). The β coefficients for biomarkers significantly associated with eGFRcr decline ranged from -0.292 to -0.098 ml/min/1.73 m^2^/year. When calculating eGFRcr using the 2021 equation, the correlation coefficient between the annual rate of change in eGFRcr-2009 and eGFRcr-2021 was 0.998 (Additional file 2: Figure S4D). Among the 233 biomarkers, 67 biomarkers were associated with eGFRcr-2021 decline (FDR < 0.05, β ranged from -0.301 to -0.104, Additional file 1: Table S7), and 65 out of these 67 were associated with eGFRcr-2009 decline. In 3 sensitivity analyses removing participants with non-fasting status, CKD, or increase in eGFRcr, 60, 62, and 63 of the 66 biomarkers remained significant, respectively (Additional file 1: Table S8 & Additional file 2: Figure S5). In sensitivity analyses model 2d (further adjusted for UACR) and 2e (inverse probability weighting-weight was applied), all 66 biomarkers remained significant, indicating that albuminuria and bias caused by loss to follow-up may not affect our associations (Additional file 1: Table S8). When investigating associations with rapid eGFRcr decline defined as the annual rate of change in eGFRcr < -3 ml/min/1.73 m^2^/year, 61 out of 66 biomarkers were positively associated with rapid eGFRcr decline, with odds ratios ranging from 1.29 to 2.09 (Additional file 1: Table S9).

When investigating their associations with the annual rate of change in eGFRcys, 38 out of 66 biomarkers were inversely associated with change in eGFRcys at levels of FDR < 0.05 (Additional file 1: Table S10 & Additional file 2: Figure S6), and associations of the total 233 biomarkers with the annual rate of change in eGFRcys are presented in Additional file 1: Table S11. Associations between baseline characteristics and the annual rate of change in eGFRcr and eGFRcys are presented in Additional file 1: Table S12.

### Associations of proteomic biomarkers with eGFRcr-based incident CKD

Among 1086 participants free of eGFRcr-based CKD at baseline, 262 cases of incident CKD were identified during 11,849 person-years of follow-up. Twenty-eight out of the 66 eGFRcr change-associated biomarkers were associated with incident CKD in model 2 (FDR < 0.05, Additional file 1: Table S10 & Additional file 2: Figure S7). The hazard ratios (HRs) for biomarkers with significant associations with incident CKD ranged from 1.16 to 1.52. The top 3 biomarkers with the highest HRs and lowest FDR for the associations were TRAIL-R2 (HR, 1.52), TNFRSF9 (HR, 1.51), and TNFRSF11A (HR, 1.49). In sensitivity analyses using the alternative definition of incident cases, 166 cases were identified and 30 out of 66 biomarkers were associated with incident CKD (FDR < 0.05, HRs ranged from 1.21 to 1.67), with 27 biomarkers overlapping with the initially identified 28 biomarkers (Additional file 1: Table S9).

**Fig. 3:**
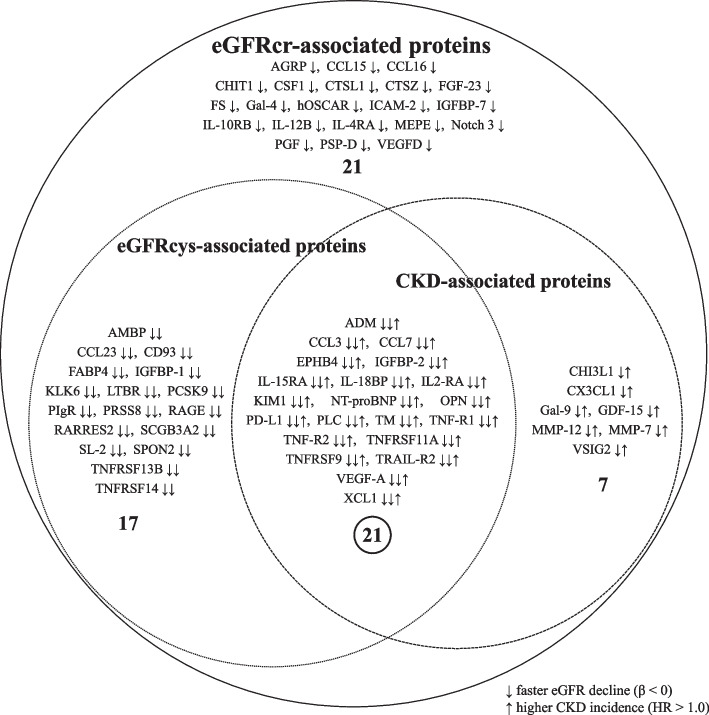
Overlap of proteomic biomarkers associated with kidney function decline and incident CKD. Kidney function decline includes both the annual rate of change in eGFRcr and the annual rate of change in eGFRcys. Detailed results are presented in Additional file 1: Table S10. Abbreviations: CKD, chronic kidney disease; eGFRcr, creatinine-based estimated glomerular filtration rate; eGFRcys, cystatin C-based estimated glomerular filtration rate; HR, hazard ratio. Full names of the biomarkers can be found in Additional file 1: Table S1

We found that 21 proteins were consistently associated with faster decline in eGFRcr, faster decline in eGFRcys, and higher risk of incident CKD (Table 2, Additional file 1: Table S10 & Fig. 3). Among these 21 biomarkers, 17 were also positively associated with higher levels of UACR at F4 (Additional file 1: Table S13). In the discovery of their potential to serve as drug targets, we found 10 out of 21 have been used as drug targets for drugs to treat a wide range of diseases or indications, and IL2-RA has been used as drug target for kidney failure and CKD treatment (Additional file 1: Table S14). All 21 identified biomarkers have been reported in previous proteomic studies (Additional file 1: Table S15). The pairwise correlations of these 21 biomarkers are shown in Additional file 2: Figure S8. After correction for multiple testing, 199 out of 210 pairs of correlations were significant, with a mean correlation coefficient of 0.35 (range 0.14 to 0.88) for significant correlations.

**Table 2:** Significant associations of 21 proteomic biomarkers with the annual rate of change in eGFR and CKD incidence ^a^

**Biomarker**	**UniProt_ID**	**Associations with change in eGFRcr** **(*N* = 1140)**			**Associations with change in eGFRcys** **(*N *= 1140)**			**Associations with CKD incidence** **(*N* = 1086; cases = 262, controls = 824)**		
		**β (95%CI)**	**P**	**FDR**	**β (95%CI)**	**P**	**FDR**	**HR (95%CI)**	**P**	**FDR**
ADM	P35318	-0.157 (-0.249, -0.065)	8.67E-04	5.31E-03	-0.138 (-0.238, -0.038)	6.78E-03	2.13E-02	1.29 (1.07, 1.55)	6.92E-03	2.28E-02
CCL3	P10147	-0.116 (-0.197, -0.036)	4.77E-03	1.98E-02	-0.101 (-0.186, -0.016)	1.98E-02	4.21E-02	1.24 (1.08, 1.42)	2.05E-03	1.13E-02
CCL7	P80098	-0.135 (-0.216, -0.053)	1.19E-03	6.91E-03	-0.095 (-0.181, -0.010)	2.88E-02	4.99E-02	1.25 (1.09, 1.44)	1.78E-03	1.12E-02
EPHB4	P54760	-0.233 (-0.313, -0.153)	1.46E-08	1.62E-06	-0.184 (-0.273, -0.095)	5.18E-05	8.57E-04	1.29 (1.10, 1.52)	2.25E-03	1.14E-02
IGFBP-2	P18065	-0.226 (-0.318, -0.135)	1.36E-06	3.65E-05	-0.168 (-0.268, -0.068)	1.01E-03	6.56E-03	1.29 (1.08, 1.54)	5.37E-03	2.07E-02
IL-15RA	Q13261	-0.181 (-0.266, -0.097)	2.59E-05	3.42E-04	-0.138 (-0.231, -0.044)	4.03E-03	1.48E-02	1.41 (1.20, 1.65)	3.13E-05	3.44E-04
IL-18BP	O95998	-0.235 (-0.317, -0.152)	2.70E-08	1.62E-06	-0.161 (-0.252, -0.069)	6.13E-04	5.06E-03	1.24 (1.05, 1.47)	1.08E-02	2.98E-02
IL2-RA	P01589	-0.194 (-0.273, -0.115)	1.66E-06	3.87E-05	-0.108 (-0.193, -0.022)	1.40E-02	3.18E-02	1.21 (1.04, 1.40)	1.26E-02	3.32E-02
KIM1	Q96D42	-0.292 (-0.377, -0.206)	4.08E-11	9.51E-09	-0.138 (-0.229, -0.047)	3.08E-03	1.32E-02	1.45 (1.23, 1.71)	9.94E-06	3.01E-04
NT-proBNP	P16860	-0.249 (-0.336, -0.162)	2.77E-08	1.62E-06	-0.238 (-0.331, -0.145)	5.66E-07	3.74E-05	1.30 (1.10, 1.54)	1.87E-03	1.12E-02
OPN	P10451	-0.156 (-0.236, -0.076)	1.37E-04	1.28E-03	-0.101 (-0.188, -0.014)	2.36E-02	4.59E-02	1.23 (1.06, 1.42)	6.15E-03	2.13E-02
PD-L1	Q9NZQ7	-0.107 (-0.187, -0.026)	9.24E-03	3.47E-02	-0.106 (-0.191, -0.022)	1.36E-02	3.18E-02	1.30 (1.13, 1.51)	3.44E-04	2.84E-03
PLC	P98160	-0.217 (-0.306, -0.128)	2.07E-06	4.38E-05	-0.142 (-0.240, -0.044)	4.58E-03	1.59E-02	1.31 (1.10, 1.57)	2.82E-03	1.33E-02
TM	P07204	-0.146 (-0.230, -0.061)	7.13E-04	4.65E-03	-0.105 (-0.195, -0.015)	2.24E-02	4.49E-02	1.26 (1.07, 1.49)	5.65E-03	2.07E-02
TNF-R1	P19438	-0.221 (-0.315, -0.127)	4.57E-06	8.88E-05	-0.174 (-0.279, -0.070)	1.09E-03	6.56E-03	1.24 (1.05, 1.47)	1.31E-02	3.33E-02
TNF-R2	P20333	-0.214 (-0.300, -0.127)	1.41E-06	3.65E-05	-0.197 (-0.294, -0.101)	6.49E-05	8.57E-04	1.31 (1.12, 1.54)	7.94E-04	5.82E-03
TNFRSF11A	Q9Y6Q6	-0.214 (-0.306, -0.121)	6.37E-06	1.14E-04	-0.208 (-0.308, -0.109)	4.37E-05	8.57E-04	1.49 (1.23, 1.79)	2.95E-05	3.44E-04
TNFRSF9	Q07011	-0.234 (-0.322, -0.145)	2.36E-07	9.18E-06	-0.170 (-0.269, -0.070)	8.59E-04	6.30E-03	1.51 (1.30, 1.75)	4.12E-08	2.72E-06
TRAIL-R2	O14763	-0.180 (-0.267, -0.094)	4.24E-05	4.94E-04	-0.105 (-0.198, -0.013)	2.61E-02	4.78E-02	1.52 (1.26, 1.84)	1.45E-05	3.01E-04
VEGF-A	P15692	-0.127 (-0.211, -0.043)	2.96E-03	1.41E-02	-0.142 (-0.230, -0.054)	1.56E-03	8.55E-03	1.27 (1.08, 1.50)	3.21E-03	1.41E-02
XCL1	P47992	-0.120 (-0.196, -0.043)	2.23E-03	1.21E-02	-0.103 (-0.183, -0.022)	1.27E-02	3.11E-02	1.16 (1.03, 1.31)	1.54E-02	3.75E-02

*Abbreviations***:** *CI* Confidence interval, *CKD* Chronic kidney disease; *eGFRcr* Creatinine-based estimated glomerular filtration rate; *eGFRcys* Cystatin C-based estimated glomerular filtration rate, *FDR* Benjamini–Hochberg false-discovery rate, *HR* Hazard ratio. Full names of the biomarkers can be found in Additional file 1: Table S1

^a^The 66 biomarkers significantly associated with the annual rate of change in eGFRcr were used to investigate their associations with the annual rate of change in eGFRcys using linear regressions and eGFRcr-based incident CKD using interval-censored Cox regressions. Detailed information and results are presented in Additional file 1: Table S10. The 21 biomarkers significantly associated with both the annual rate of change in eGFRcys and incident CKD (FDR < 0.05) are presented in this table

### Mendelian randomization analysis

The MR analysis results show a potential causal association of a higher level of TNFRSF11A (β = 0.024, *P*-value = 0.030) with faster eGFR decline (β > 0 means a positive association with eGFR decline [32]). However, no significant associations were observed for any of the proteins after multiple corrections (FDR > 0.05, Table 3).

**Table 3:** Results of the two-sample Mendelian randomization analysis between 17 proteins and eGFR decline

**Biomarkers**	**MR_Method**	**No. IVs**	**% variance explained by SNP**^**a**^	**β (95%CI)**	**SE**	***P*-value**	**FDR**
ADM	Wald ratio	1	1.32	0.033 (-0.026, 0.092)	0.030	0.279	0.694
CCL7	Wald ratio	1	0.61	-0.022 (-0.105, 0.061)	0.042	0.610	0.845
EPHB4	Wald ratio	1	1.23	-0.015 (-0.070, 0.041)	0.028	0.609	0.845
IGFBP-2	Wald ratio	1	0.20	0.008 (-0.133, 0.150)	0.072	0.908	0.965
IL-15RA	Wald ratio	1	2.92	-0.028 (-0.065, 0.010)	0.019	0.147	0.626
IL2-RA	Wald ratio	1	14.8	-0.004 (-0.022, 0.013)	0.009	0.646	0.845
OPN	Wald ratio	1	0.44	0.065 (-0.035, 0.165)	0.051	0.202	0.687
PD-L1	Wald ratio	1	4.61	0.010 (-0.018, 0.038)	0.014	0.488	0.829
PLC	Wald ratio	1	1.27	-0.054 (-0.110, 0.002)	0.029	0.058	0.463
TM	Wald ratio	1	1.21	-0.008 (-0.062, 0.046)	0.028	0.767	0.884
TNF-R1	Wald ratio	1	0.31	0.020 (-0.122, 0.162)	0.072	0.780	0.884
TNF-R2	Wald ratio	1	1.24	0.022 (-0.029, 0.073)	0.026	0.394	0.744
TNFRSF11A	Wald ratio	1	11.2	0.024 (0.002, 0.046)	0.011	**0.030**	0.463
TNFRSF9	Wald ratio	1	1.21	0.048 (-0.006, 0.103)	0.028	0.082	0.463
TRAIL-R2	Wald ratio	1	5.58	-0.001 (-0.028, 0.027)	0.014	0.968	0.968
VEGF-A	Wald ratio	1	24.2	0.006 (-0.006, 0.019)	0.006	0.317	0.694
XCL1	Wald ratio	1	22.4	0.006 (-0.006, 0.019)	0.006	0.327	0.694

*Abbreviations*: *CI* Confidence interval, *eGFR* Glomerular filtration rate, *IVs* Instrumental variables, *FDR* Benjamini–Hochberg false-discovery rate, *MR* Mendelian randomization, *SE* Standard error, *SNP* Single nucleotide polymorphism. Full names of the biomarkers can be found in Additional file 1: Table S1

^a^The proportion of proteomic biomarkers’ variance explained by the SNP [31]

### Pathway enrichment analysis

A total of 254 pathways that reached statistical significance (FDR < 0.05) were identified when using 21 biomarkers (Table 2 & Fig. 3). The top 15 enriched pathways were characterized by processes relating to the response of a tumor necrosis factor (TNF) stimulus, T cell proliferation, monocyte chemotaxis, and regulation of lymphocyte and leukocyte chemotaxis (Table 4 & Additional file 2: Figure S9).

**Table 4:** Pathway enrichment analysis of the 21 identified biomarkers showing top biological processes related to kidney function ^a^

**Gene Ontology**	**Description**	**Gene**	**FDR**
GO:0071356	Cellular response to tumor necrosis factor	TNFRSF11A, XCL1, CCL3, CCL7, TNFRSF9, TNFRSF1A, TNFRSF1B	2.01E-05
GO:0034612	Response to tumor necrosis factor	TNFRSF11A, XCL1, CCL3, CCL7, TNFRSF9, TNFRSF1A, TNFRSF1B	2.01E-05
GO:0035747	Natural killer cell chemotaxis	XCL1, CCL3, CCL7	7.27E-05
GO:0002548	Monocyte chemotaxis	TNFRSF11A, XCL1, CCL3, CCL7	1.87E-04
GO:0042129	Regulation of T cell proliferation	XCL1, CD274, IGFBP2, IL2RA, TNFRSF1B	1.87E-04
GO:0032496	Response to lipopolysaccharide	ADM, THBD, TNFRSF11A, CCL3, CD274, TNFRSF1B	1.87E-04
GO:0007565	Female pregnancy	ADM, THBD, VEGFA, IGFBP2, SPP1	1.87E-04
GO:0140131	Positive regulation of lymphocyte chemotaxis	XCL1, CCL3, CCL7	1.87E-04
GO:0002237	Response to molecule of bacterial origin	ADM, THBD, TNFRSF11A, CCL3, CD274, TNFRSF1B	1.95E-04
GO:0042098	T cell proliferation	XCL1, CD274, IGFBP2, IL2RA, TNFRSF1B	2.21E-04
GO:0050727	Regulation of inflammatory response	TNFRSF11A, XCL1, CCL3, IL2RA, TNFRSF1A, TNFRSF1B	2.21E-04
GO:0044706	Multi-multicellular organism process	ADM, THBD, VEGFA, IGFBP2, SPP1	2.29E-04
GO:0002690	Positive regulation of leukocyte chemotaxis	XCL1, CCL3, CCL7, VEGFA	2.29E-04
GO:1,901,623	Regulation of lymphocyte chemotaxis	XCL1, CCL3, CCL7	2.29E-04
GO:0097529	Myeloid leukocyte migration	TNFRSF11A, XCL1, CCL3, CCL7, VEGFA	2.29E-04

*Abbreviations*: *CKD* Chronic kidney disease, *eGFRcr* Creatinine-based estimated glomerular filtration rate, *eGFRcys* Cystatin C-based estimated glomerular filtration rate, *FDR* Benjamini–Hochberg false-discovery rate

^a^The 21 biomarkers significantly associated with the annual rate of change in eGFRcr, the annual rate of change in eGFRcys, and incident CKD (Table 2 & Fig. 3), were included in the pathway enrichment analysis. The y-axis signifies the top 15 biological processes in kidney function. The x-axis is the -log10 of the FDR

## Discussion

In this prospective cohort study, we investigated the associations of 233 proteins with longitudinal change in kidney function and incident CKD among 1140 participants. A total of 66 biomarkers were associated with the annual rate of change in eGFRcr in discovery analysis, and 21 biomarkers out of these, were found to be also associated with both the annual rate of change in eGFRcys and incident CKD. Using a two-sample MR approach, we provided preliminary evidence suggesting a potential causal association between TNFRSF11A and kidney function decline (*P*-value = 0.030, FDR = 0.463).

All 21 biomarkers that we identified were associated with greater kidney disease risk, probably because we targeted 233 CVD- and inflammation-related biomarkers. Our results were consistent with previous studies investigating proteomic biomarkers measured by the same Olink panels and kidney function [8, 12, 15]. Some of the proteins that we identified are well-known biomarkers of kidney function, such as kidney injury molecule (KIM1), TNF-R1, TNF-R2, and TNF-related apoptosis-inducing ligand receptors 2 (TRAIL-R2), supporting the feasibility of proteomic analysis to identify biomarkers of kidney function decline. In the present study, KIM1 was the biomarker with the strongest association with kidney function decline. KIM1 has been extensively studied and represents a potential biomarker of tubular injury in both animals and humans [39, 40]. Longitudinal studies have also reported that urinary and blood KIM1 are positively associated with kidney function decline, incident CKD, and CKD progression in both diabetic patients and the general population [8, 13, 14, 39, 41, 42]. When investigating the associations with incident CKD, TRAIL-R2 was the biomarker with the strongest association in our study. The best-understood function of TRAIL-R2 is the induction of apoptosis [43]. TRAIL-R2 has been found to be associated with kidney function decline in several proteomic studies [8, 10, 11, 13]. For example, TRAIL-R2 was the biomarker with the strongest association with kidney function decline among 80 CVD-related plasma proteins in a longitudinal study [10]. Several other TNF superfamily receptors (TNFRSF) were found to be related to kidney function decline in our study, including TNF-R1, TNF-R2, TNFRSF9, and TNFRSF11A. In our pathway enrichment analysis, TNF response- and inflammatory response-related pathways were in the top 15 pathways related to kidney function and CKD (Table 4 & Additional file 2: Figure S9). Thus, our study provides additional evidence that TNF signaling pathways and inflammation may play a role in the pathophysiology of CKD. Similarly, a previous study identified a panel of 17 proteins from 194 plasma inflammatory proteins to be associated with end-stage kidney disease (ESKD) risk in diabetic patients, and these 17 proteins were enriched for TNF superfamily receptors [9]. The findings supported the involvement of immune response mechanisms in the development of CKD, which is also consistent with our pathway analysis, implicating T cell proliferation-related mechanisms involved in kidney function pathophysiology. Three chemokines, including C–C motif chemokine 3 (CCL3), C–C motif chemokine 7 (CCL7), and lymphotactin (XCL1), were involved in more than half of the top 15 pathways, and have been reported to be inversely associated with kidney function in previous studies [8, 10, 12, 13, 15]. Chemokines may play a key role in guiding inflammatory cells into the sites of inflammation in kidneys and recruiting immune cells such as T cells and monocytes during the development of chronic kidney injury [44]. Increasing evidence suggests that chemokines and their receptors may be potential targets for anti-inflammatory therapy in CKD [45].

Another important biomarker we identified was N-terminal prohormone brain natriuretic peptide (NT-proBNP), which has been shown to be a reliable biomarker for diagnosis of heart failure and prognostic evaluation among patients with heart failure [46]. In the present study, NT-proBNP was the second strongest biomarker associated with kidney function decline, which is in line with findings from other KORA cohort-based studies [47, 48]. Similarly, a recent proteomic study found that higher plasma NT-proBNP was associated with worsening kidney function among 5131 patients with type 2 diabetes [8]. Community-based longitudinal studies have also found that blood NT-proBNP is positively associated with kidney function decline and incident CKD [49, 50]. The exact mechanisms explaining the link between NT-proBNP and kidney function decline remain unclear. Several pathways have been proposed to explain the association. The increase in blood NT-proBNP can result from cardiac stretch, volume overload, and venous congestion, which in turn, are potential risk factors of kidney function decline. For example, volume overload or venous congestion can lead to an increase in central venous pressure, which has been reported to be associated with impaired kidney function [51, 52]. On the other hand, NT-proBNP is partially dependent on kidney clearance for elimination, so the concentration of NT-proBNP accumulates with impaired kidney function [53, 54]. Thus, NT-proBNP could be only a marker for other kidney-damaging risk factors rather than a causal risk factor itself. Further studies are warranted to explore the underlying mechanisms. In addition to NT-proBNP, we also identified another heart failure-related biomarker, adrenomedullin, which has previously been found to play a pathophysiological role in kidney disease [55].

A similar previous study by Grams et al. [14] investigated associations of 4877 proteins measured by the SomaScan platform with a composite outcome of more than 50% eGFR decline or ESKD among 3 American-based cohorts, including 2 kidney disease-related cohorts (1 of them was an African American cohort). However, our study was based on a community-based cohort of a relatively healthy European population. As differences in dietary habits and genetic background between ethnically diverse populations may affect both levels of protein expression and kidney function, it is important to verify observed associations in independent populations from various regions. Furthermore, most of the CKD cases we identified did not yet progress to ESKD (only 2 out of 262 incident cases had ESKD, i.e., eGFRcr < 15 ml/min per 1.73m^2^). Thus, we mainly focused on the annual rate of kidney function decline as an outcome rather than severe eGFR decline (e.g., ≥ 50% decline) or ESKD. Of note, our sensitivity analyses on rapid kidney function decline and redefinition of incident CKD cases show robust results (Additional file 1: Table S9). In our study, we targeted 233 CVD- and inflammation-related proteins measured by the Olink platform based on prior knowledge of close CKD-CVD relations, providing a more targeted approach to uncover pathways and mechanisms underlying kidney disease compared to the more comprehensive SomaScan platform used in the previous study [14]. However, it is worth noting that the smaller number of proteins measured by the Olink platform may be seen as a disadvantage compared to the SomaScan platform [56]. In a study by Rooney et al. [57] comparing correlations of 417 proteins that overlapped between the Olink and SomaScan platforms in 427 participants, the median Spearman correlation coefficient was 0.53 (range -0.21 to 0.97) and only 19% of the correlation coefficients were higher than 0.8. When Rooney et al. [57] further investigated associations of the overlapping proteins with eGFR, Olink platform-based proteins demonstrated more associations with eGFR, particularly in the group of proteins with Spearman correlation coefficients less than 0.3. Katz et al. [56] reported similar results and showed that the median Spearman correlation coefficient of proteins that overlapped between the two platforms was 0.45. These results show that findings from proteomic studies can be affected by the used platform, but the superiority of one platform over the other has not been clearly established yet. Thus, proteomic studies based on different platforms are important and our study adds to the existing literature in the field.

In MR analysis, our results show preliminary evidence suggesting a potential causal association of TNFRSF11A with eGFR decline (*P*-value = 0.030). A previous GWAS in 583 coronary patients observed an association of a polymorphism located within the genomic region of TNFRSF11A with kidney function decline [58]. In line with these associations, longitudinal studies that examined proteomics of kidney function also reported that plasma TNFRSF11A was positively associated with kidney function [8, 11]. MR is an effective approach to provide a robust and less biased estimate of causal associations, but our MR analysis was limited by the availability of GWAS, especially GWAS of proteins measured using the Olink platform. As GWAS by Sun et al. [31] reported only cis-SNPs with significance at the level *p* < 3.4E-11, each protein had only 1 cis-SNP as instrument, which limited the possibility to test the robustness of our results in sensitivity analyses and may have reduced our statistical power [59]. Additionally, the presence of overlapping participants in the 2 GWAS used in the present MR analysis may have caused bias [35], although this potential bias did not significantly change our MR results when using a maximum likelihood method to address it (details in Additional file 2: Text S3 & Additional file 1: Table S16). Further MR analyses based on multiple instruments GWAS summary statistics from larger populations are warranted.

Study strengths include the assessment of a large number of proteomic biomarkers and the use of a large prospective cohort study, with a median follow-up time of 13.4 years and two follow-up visits for most participants. The Strengthening the Reporting of Observational Studies in Epidemiology checklists for cohort studies and MR studies are presented in Additional file 1: Table S17 & 18, respectively. However, several limitations should also be considered. First, we used FDR to correct for multiple testing, which is less conservative and may increase the risk of false positives compared to Bonferroni correction. Furthermore, we did not validate the identified biomarkers in external cohorts since all our 21 identified biomarkers have been reported in previous proteomic studies (Additional file 1: Table S15). Second, there may be misclassification resulting from measurement errors due to different measurement methods of serum creatinine and cystatin C at S4/F4/FF4, and imputation of missing value of eGFRcys. Additionally, we defined CKD cases based on a single creatinine measurement, which does not fulfill the diagnosis of CKD in clinical practice that the presence of eGFRcr < 60 ml/min per 1.73m^2^ persists for more than 3 months. Thus, because our data may not be ideally suited for predictive analysis due to various limitations, such as a suboptimal CKD diagnosis and lack of external validation cohorts, we did not develop a prediction model for the development and progression of CKD. Our primary aim was to identify potential biomarkers related to kidney function decline, which may contribute to the development of predictive models, diagnostic strategies, and therapeutic targets in the future. Third, there may be selection bias due to loss to follow-up, but the results of sensitivity analysis with inverse probability weights suggest that this may not affect our result remarkably. Finally, although we adjusted for multiple confounders, we were unable to adjust for albuminuria at baseline due to lack of data.

## Conclusions

In conclusion, we found 21 known proteins to be associated with kidney function decline and incident CKD in a Caucasian community-based population and provided further evidence regarding new diagnostic or prognostic biomarkers and therapeutic targets for CKD. Although the current underpowered MR analysis failed to find convincing evidence for causal associations of the 21 proteins with kidney function decline, our results provide preliminary evidence suggesting a potential causal association between TNFRSF11A and kidney function decline and a role of TNF signaling pathways in the pathophysiology of CKD. Further MR studies are needed to establish and validate a conclusive causal association.

## Supplementary Information

**Additional file 1:** **Table S1.** Normalized expressionvalues for proteomic biomarkers at baseline; **Table S2.** SNPs-traitsassociations retrieved from PhenoScanner V2 for proteins-related SNPs; **Table S3.** Harmonized summary statisticsused in Mendelian randomization analysis; **TableS4.** Characteristics of participants over the study period; **Table S5.** Baseline characteristics forparticipants with and without follow-up information on eGFRcr; **Table S6.** Longitudinal associationsbetween 233 proteomic biomarkers and the annual rate of change in eGFRcr; **Table S7.** Longitudinal associations between 233 proteomicbiomarkers and the annual rate of change in CKD-EPI Equation 2009-based andCKD-EPI Equation 2021-based eGFRcr;**Table S8.** Sensitivity analyses oflongitudinal associations between 66 proteomic biomarkers and the annual rateof change in eGFRcr; **Table S9.** Sensitivityanalyses of association of 66 proteomic biomarkers with rapid decline in eGFRcrand CKD incidence; **Table S10.** Associationof 66 proteomic biomarkers with the annual rate of change in eGFRcys and CKDincidence; **Table S11.** Longitudinalassociations between 233 proteomic biomarkers and the annual rate of change ineGFRcys; **Table S12.** Longitudinal associationsbetween baseline characteristics and the annual rate of change in eGFRcr andeGFRcys; **Table** **S13.** Association of 21 proteomic biomarkers with UACR; **Table S14.** Summary of druggable targetsand their corresponding disease of the 21 identified proteins; **Table S15.** Proteomic biomarkersassociated with kidney function and/or chronic kidney disease in previouscross-sectional or longitudinal proteomic studies; **Table S16.** Evaluation of bias due to participant overlap intwo-sample Mendelian randomization analysis; **Table S17.** STROBEStatement—checklist of items in reports of observational studies; **Table S18.** STROBE-MR checklist of recommended items in reports of Mendelianrandomization studies.

**Additional file 2:** **Text S1.** Assessment of kidneyoutcomes; **TextS2.** Inverse probabilityweighting; **Text S3.** Mendelianrandomization analysis;** Figure S1.** Exampleof the annual rate of change in eGFR for each participant; **Figure S2.** Flowchart of statistical analyses; **Figure S3.** Genetic instrument selection and data harmonization forMendelian randomization analysis; **FigureS4.** Distribution and correlation between the annual rate of change ineGFRcr and eGFRcys; **Figure S5.** Overlapof proteomic biomarkers between biomarkers associated with the annual rate ofchange in eGFRcr in several sensitivity analyses; **Figure S6.** Longitudinal associations between 66 proteomicbiomarkers and the annual rate of change in eGFRcys; **Figure S7.** Association of 66 proteomic biomarkers with eGFRcr-basedCKD incidence; **Figure S8.** Pairwisecorrelation matrix between the 21 identified proteomic biomarkers; **Figure S9.** Pathway enrichment analysisof the 21 identified biomarkers showing top biological processes related tokidney function.

## Data Availability

The informed consent given by KORA study participants does not cover data posting in public databases. Cooperation partners can obtain permission to use KORA data under the terms of a project agreement (https://helmholtz-muenchen.managed-otrs.com/external).

## Abbreviations

CKD: Chronic kidney disease
CVD: Cardiovascular disease
eGFR: Estimated glomerular filtration rate
eGFRcr: Creatinine-based estimated glomerular filtration rate
eGFRcys: Cystatin c-based estimated glomerular filtration rate
ESKD: End-stage kidney disease
FDR: Benjamini–Hochberg false-discovery rate
GWAS: Genome-wide association study
HR: Hazard ratio
KORA: Cooperative Health Research in the Region of Augsburg
MR: Mendelian randomization
SD: Standard deviation
SNP: Single nucleotide polymorphism
TNF: Tumor necrosis factor
UACR: Urine albumin-creatinine ratio
