# Supplementary material for: Proteomic profiling of longitudinal changes in kidney function among middle-aged and older men and women: the KORA S4/F4/FF4 study
Source: BMC Med. 2023 Jul 5;21:245. doi: 10.1186/s12916-023-02962-z (PMC10324145; doi:10.1186/s12916-023-02962-z)

Additional file 2: Supplementary Text and Figure

Proteomic profiling of longitudinal changes in kidney function among middle-aged and older men and women: the KORA S4/F4/FF4 Study

Jie-sheng Lin, Jana Nano, Agnese Petrera, Stefanie M. Hauck, Tanja Zeller, Wolfgang Koenig, Christian L. Müller, Annette Peters, Barbara Thorand

Contents

Text S1. Assessment of kidney outcomes 3
Text S2. Inverse probability weighting 4
Text S3. Mendelian randomization analysis 5
Figure S1. Example of the annual rate of change in eGFR for each participant 7
Figure S2. Flowchart of statistical analyses 8
Figure S3. Genetic instrument selection and data harmonization for Mendelian randomization analysis 9
Figure S4. Distribution and correlation between the annual rate of change in eGFRcr and eGFRcys 10
Figure S5. Overlap of proteomic biomarkers between biomarkers associated with the annual rate of change in eGFRcr in several sensitivity analyses 11
Figure S6. Longitudinal associations between 66 proteomic biomarkers and the annual rate of change in eGFRcys. 12
Figure S7. Association of 66 proteomic biomarkers with eGFRcr-based CKD incidence 13
Figure S8. Pairwise correlation matrix between the 21 identified proteomic biomarkers 14
Figure S9. Pathway enrichment analysis of the 21 identified biomarkers showing top biological processes related to kidney function 15

Text S1. Assessment of kidney outcomes

Cystatin C was measured using the ARCHITECT MULTIGENT Cystatin C assay (Abbott, Wiesbaden, Germany) using immunoturbidimetry at S4 (baseline) and N Latex Cystatin C assay (Siemens Healthcare Diagnostics Products GmbH) using particle-enhanced immunonephelometry at F4/FF4 (first and second follow-up). Cystatin C at F4 was measured during July-October 2008, so the values were calibrated to the International Federation of Clinical Chemistry and Laboratory Medicine (IFCC) standard by multiplying by a factor of 1.174 as suggested by Siemens. S4 and FF4 values were measured in 2015 and 2017, respectively, and corresponded to the IFCC standard. Cystatin C-based estimated glomerular filtration rate (eGFRcys) was calculated using the Chronic Kidney Disease Epidemiology Collaboration equation 2012 [23].

In comparison to creatinine-based estimated glomerular filtration rate (eGFRcr), there were 278, 5, and 17 missing values on eGFRcys at S4, F4, and FF4, respectively. Regression imputation was used to impute the missing values of eGFRcys. A linear mixed-effects model with random intercepts for each participant was constructed as eGFRcys (dependent variable) against eGFRcr (independent variable), and included age at the time of eGFR measurement, sex, and follow-up wave as covariates, using R package “lme4”. The predicted values were used to replace the missing values on eGFRcys.

Additionally, at KORA F4, urinary albumin and urinary creatinine were determined from frozen urine (sampled by a random spot urine specimen) with a modified kinetic rate Jaffe method (CREATININ-JK, Greiner, Bahlingen, Germany) on a Cobas Mira analyzer (Roche Diagnostics, Mannheim, Germany) and by nephelometry on a BN II analyzer (Siemens, Erlangen, Germany). Urinary albumin to creatinine ratio was calculated as urinary albumin/urinary creatinine (mg/g).

Text S2. Inverse probability weighting

To partially address bias caused by loss to follow-up (due to death or other reasons, e.g., refusal or inability to contact, Figure 1B), the inverse probability weighting method [27] was used to examine the impact of loss to follow-up in the present study. Each participant's probability of loss to follow-up (P1) was estimated by logistic regression model with loss to follow-up (yes/no) as outcomes, including baseline age, sex, body mass index, physical activity, smoking status, alcohol consumption, systolic blood pressure, use of antihypertensive medication, triglycerides (naturally log-transformed), high-density lipoprotein cholesterol, use of lipid-lowering medication, prevalent diabetes, prevalent cardiovascular diseases, fasting status, and creatinine-based estimated glomerular filtration rate (eGFRcr) as predictors. Inverse probability weighting-weight was calculated as 1/(1-P1). Then the weight was applied in sensitivity analyses of associations between the 66 significant biomarkers and the annual rate of change in eGFRcr in linear regression models.

Text S3. Mendelian randomization analysis

Capitalizing on the fact that genotypes are assigned randomly when passed from parents to offspring, Mendelian randomization (MR) represents an effective approach to reducing the risk of reverse causation by employing single nucleotide polymorphisms (SNPs) as instrumental variables (IV) to assess unconfounded exposure-outcome associations. To maximize statistical power, we applied a two-sample MR design using the largest genome-wide association studies (GWAS) results to date for selecting instruments for our significant biomarkers and kidney function decline. Additional file 2: Figure S3 shows the process of MR analysis. Publicly available protein quantitative trait loci (pQTL) data for 1463 Olink Explore 1536-based proteins (also based on the Olink's proximity extension assay technology) in 35571 European-ancestry population from UK Biobank Pharma Proteomics Project [31] was used to identify SNPs associated with eGFR decline-associated proteins. We found pQTLs for all 21 top proteins. Selection of pQTL was based on cis-SNPs with a multiple-testing-corrected significance at the level $p < 3.4E-11$ (associations with a genome-wide significance, i.e., $p < 5.0E-8$, are unavailable). In the next step, linkage disequilibrium clumping with $r^2 < 0.001$ within 10000kb region was conducted to identify independent SNPs. Since only 1 SNP was available for each protein, none of the SNPs was excluded in this step. SNPs-eGFR decline associations were extracted from a Meta-analysis, including 62 European-ancestry GWAS in 343339 individuals, which aimed to identify genetic loci for eGFR decline, which was defined as "(eGFR at follow-up – eGFR at baseline)/ number of years of follow-up" [32]. Eighteen out of 21 proteins had SNPs-eGFR decline data available. Furthermore, we conducted data harmonization to make sure that the effects of SNPs on proteins and eGFR decline were corresponding to the same allele. In order to test the assumption of MR that IVs should not be associated with confounders, associations between selected SNPs and other traits were searched for in the PhenoScanner V2 [33]. One SNP (rs198389) was excluded given its associations with blood pressure (Additional file 1: Table S2), leaving 17 proteins for MR analysis (Additional file 1: Table S3). Wald ratio

was calculated since only one SNP instrument was available for each protein. All the MR analyses were performed using R package “TwoSampleMR v.0.5.6” [34].

There may be participant overlap between the two GWAS studies, since the GWAS of proteins was conducted among 35571 participants from UK Biobank and the GWAS of eGFR decline was conducted among 343339 European-ancestry individuals, including 15442 participants from UK Biobank. Thus, there may be bias due to the participant overlap [35]. A maximum likelihood method was used to address this problem, using R package “MendelianRandomization v.0.6.0”. The function “MendelianRandomization::mr_maxlik” allows setting a correlation parameter *psi*, which indicates the correlation between the association with the exposure and the association with the outcome for each variant resulting from sample overlap [36], to adjust for bias due to the participant overlap in two-sample MR analysis. The parameter *psi* is set to zero when there is no participant overlap, and arises if the samples for the associations with the exposure and the outcome overlap. Because we were unable to estimate the exact correction parameter *psi* among overlapping samples, several values for *psi* (0.1, 0.3, and 0.5) were set to evaluate the bias due to participant overlap.

The STROBE (Strengthening the Reporting of Observational Studies in Epidemiology)-MR checklist of recommended items to address in reports of MR studies [37] is presented in Additional file 1: Table S18.

Figure S1. Example of the annual rate of change in eGFR for each participant

Abbreviations: eGFR, estimated glomerular filtration.

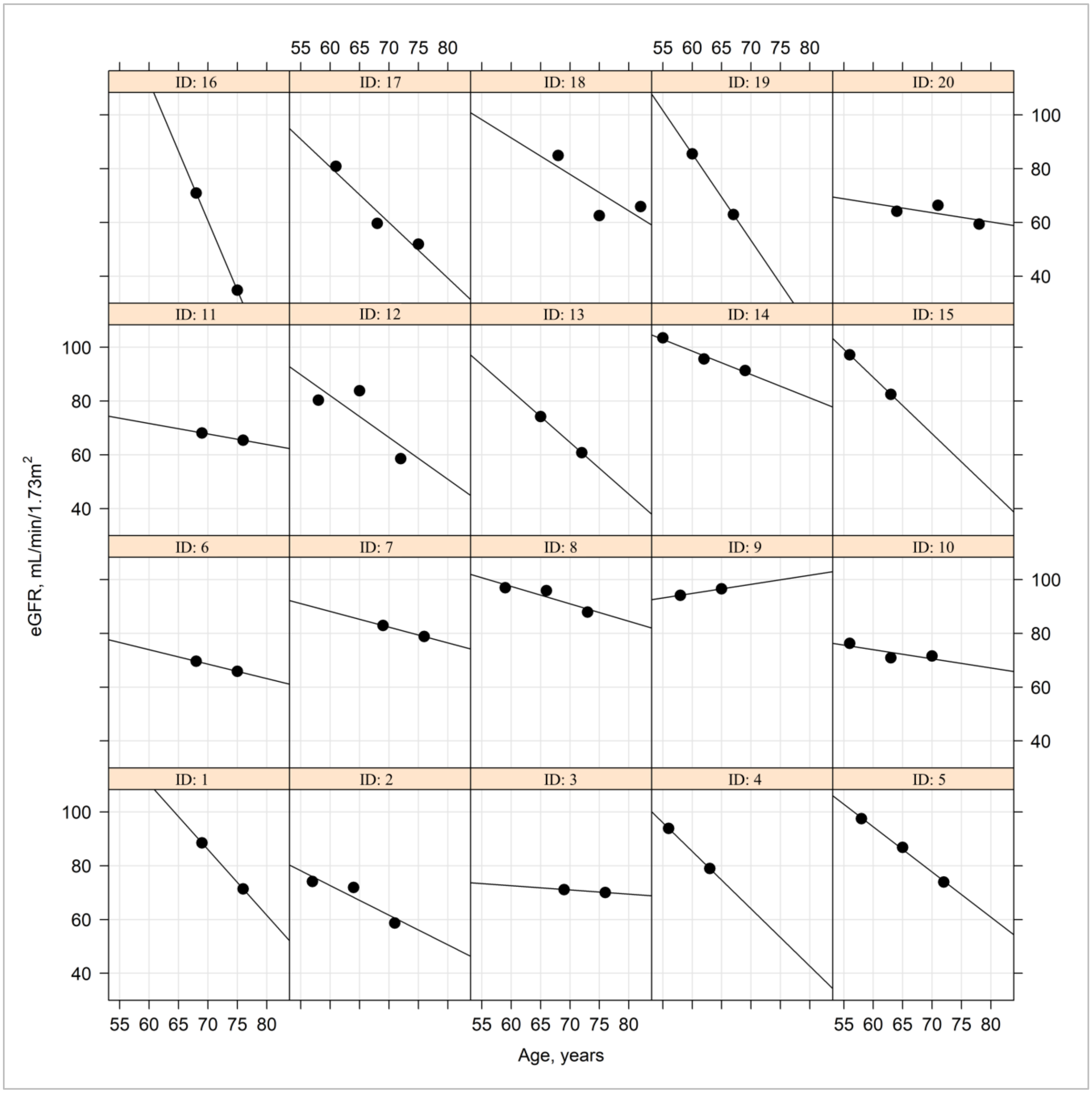

Figure S2. Flowchart of statistical analyses

Abbreviations: CKD, chronic kidney disease based on eGFRcr; eGFRcr, creatinine-based estimated glomerular filtration rate; eGFRcys, cystatin C-based estimated glomerular filtration rate; FDR, Benjamini–Hochberg false-discovery rate.

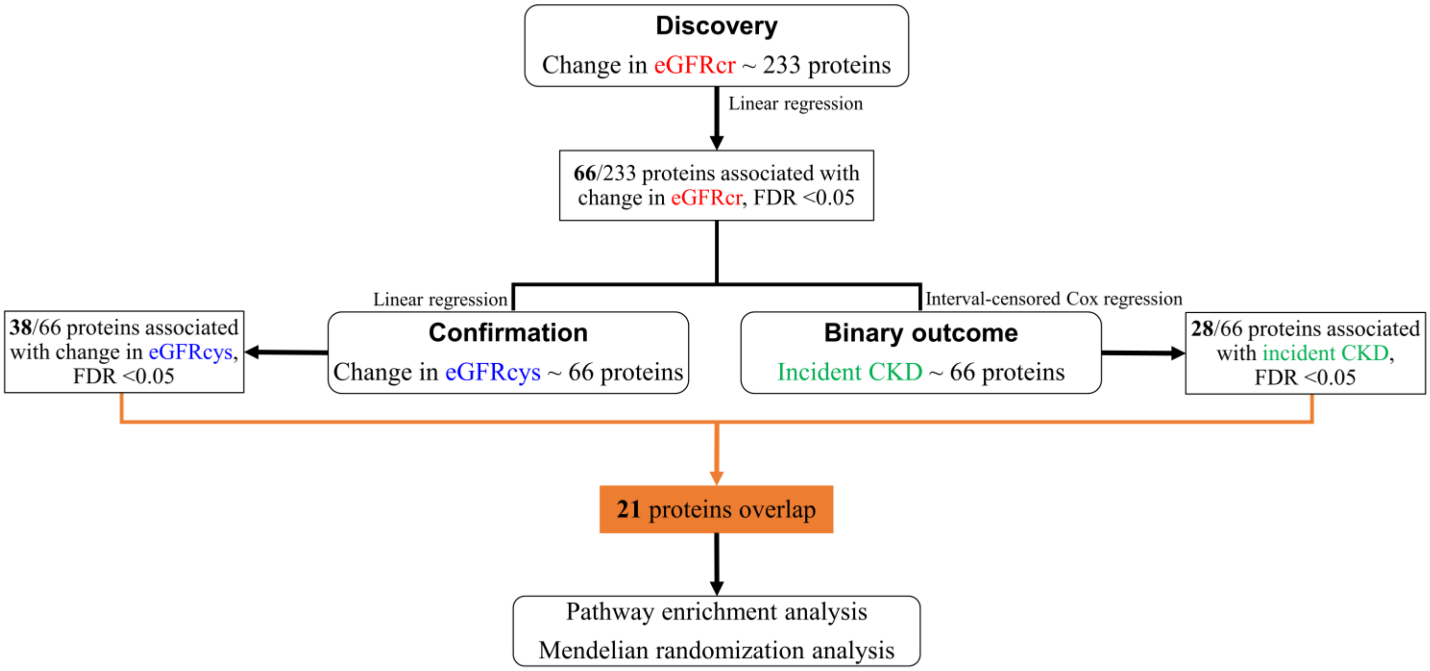

Figure S3. Genetic instrument selection and data harmonization for Mendelian randomization analysis

Abbreviations: eGFR, glomerular filtration rate; LD, linkage disequilibrium; MR, Mendelian randomization; SNP, single nucleotide polymorphism.

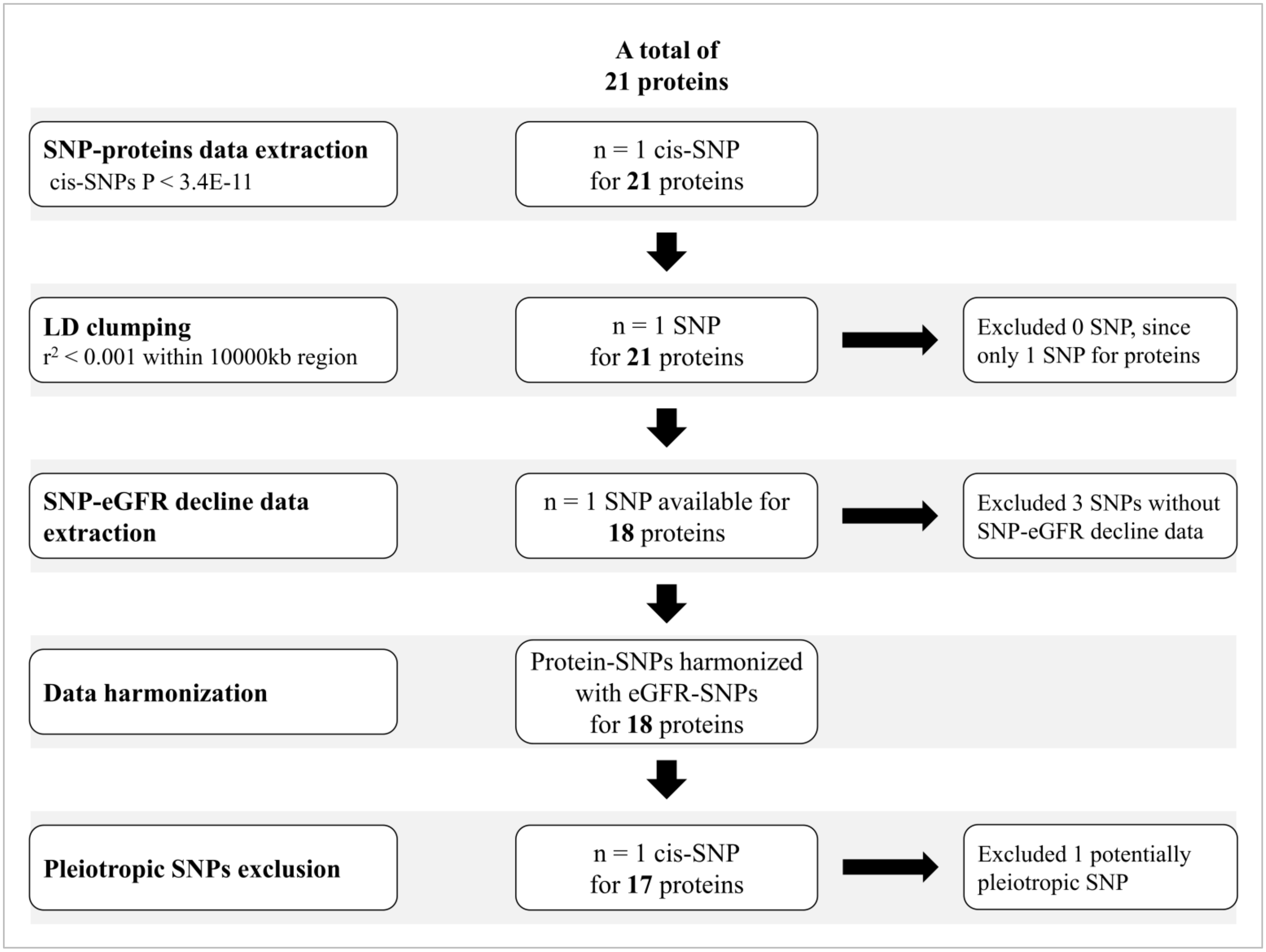

Figure S4. Distribution and correlation between the annual rate of change in eGFRcr and eGFRcys. A) Distribution of the annual rate of change in eGFRcr. **B)** Distribution of the annual rate of change in eGFRcys. **C)** Correlation between the annual rate of change in eGFRcr and eGFRcys. **D)** Correlation between the annual rate of change in eGFRcr and eGFRcr 2021.

Abbreviations: eGFRcr, creatinine-based estimated glomerular filtration rate; eGFRcr 2021, eGFRcr calculated by the Chronic Kidney Disease Epidemiology Collaboration equation 2021; eGFRcys, cystatin C-based estimated glomerular filtration rate; No.Measurement, the number of measurements on eGFR at baseline and follow-up.

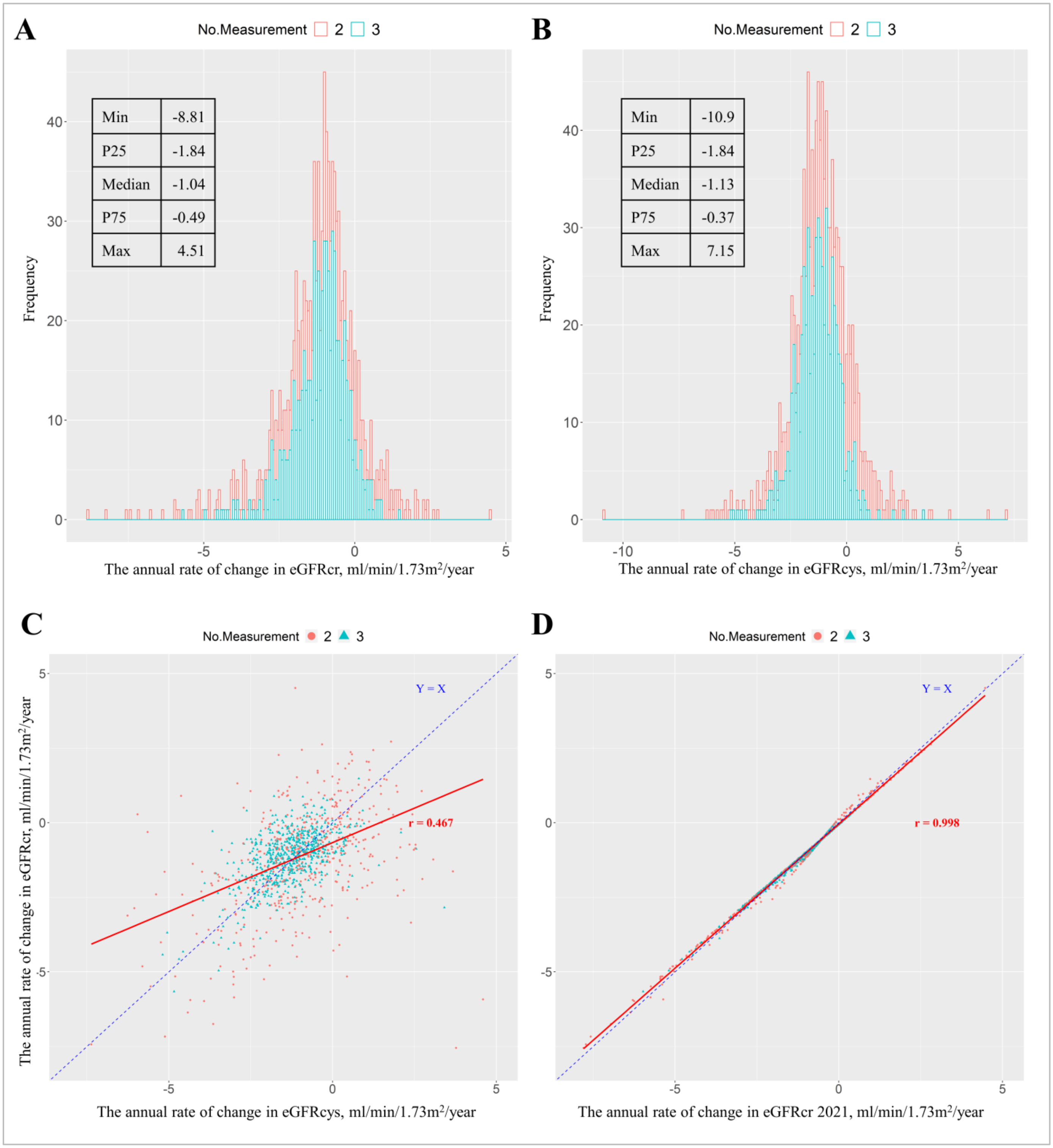

Figure S5. Overlap of proteomic biomarkers between biomarkers associated with the annual rate of change in eGFRcr in several sensitivity analyses. Several sensitivity analyses were performed based on model 2 in Additional file 1: Table S6, and only the 66 biomarkers significantly associated with the annual rate of change in eGFRcr were included. Detailed information is presented in Additional file 1: Table S8. Model 2a: repeated analyses of model 2 after excluding participants who were non-fasting before at the time of blood sampling (n = 113); Model 2b: repeated analyses of model 2 after excluding participants who had chronic kidney disease at baseline (n = 54); Model 2c: repeated analyses of model 2 after excluding participants who had an increase in eGFRcr during follow-up (n = 151).

Abbreviations: eGFRcr, creatinine-based estimated glomerular filtration rate.

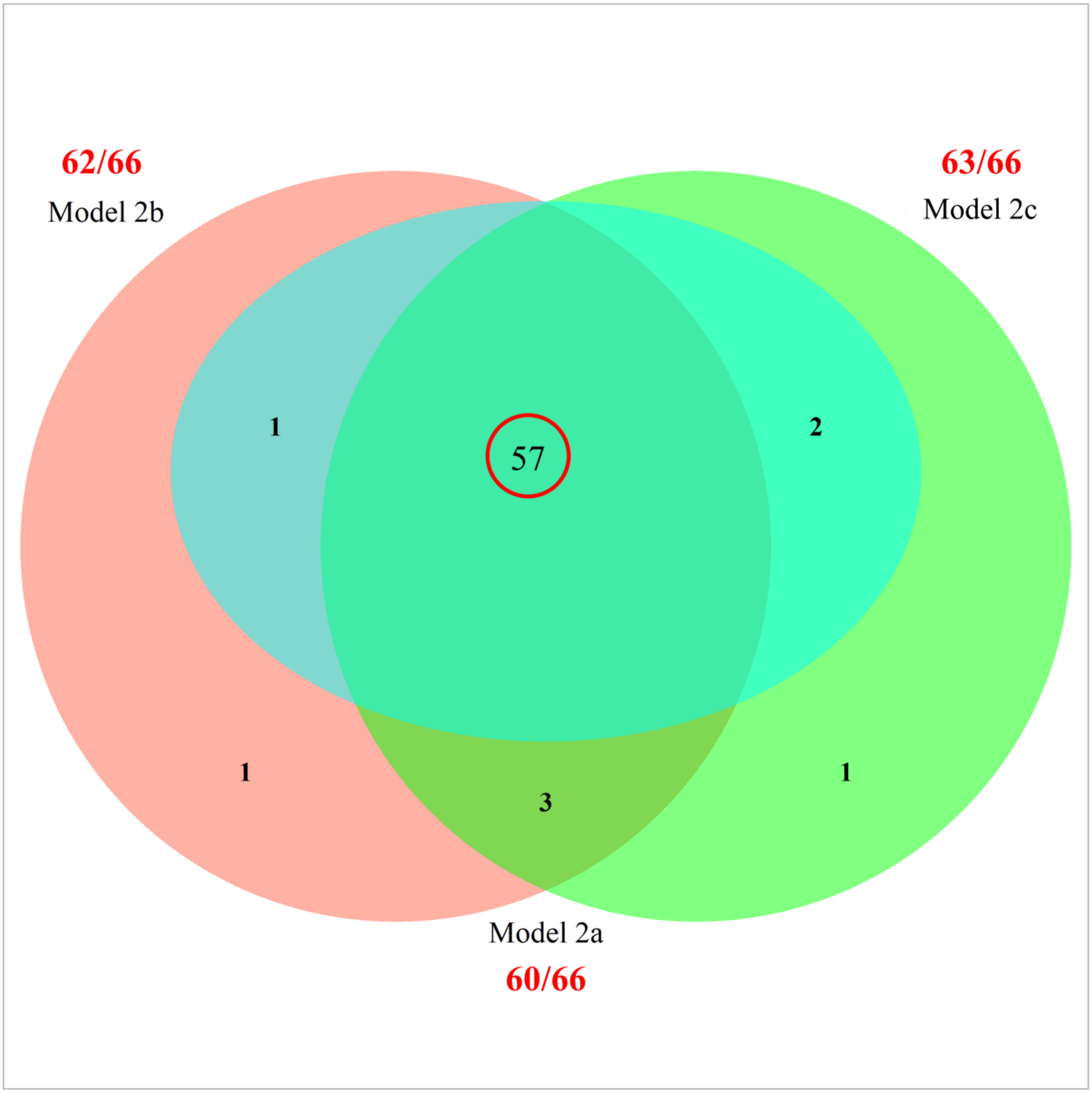

Figure S6. Longitudinal associations between 66 proteomic biomarkers and the annual rate of change in eGFRcys. The 66 biomarkers significantly associated with the annual rate of change in eGFRcr were taken to investigate their associations with the annual rate of change in eGFRcys. Detailed results of beta coefficients and FDR for the associations are presented in Additional file 1: Table S9.

Abbreviations: eGFRcr, creatinine-based estimated glomerular filtration rate; eGFRcys, cystatin C-based estimated glomerular filtration rate; FDR, Benjamini–Hochberg false-discovery rate. Full names of the biomarkers can be found in Additional file 1: Table S1.

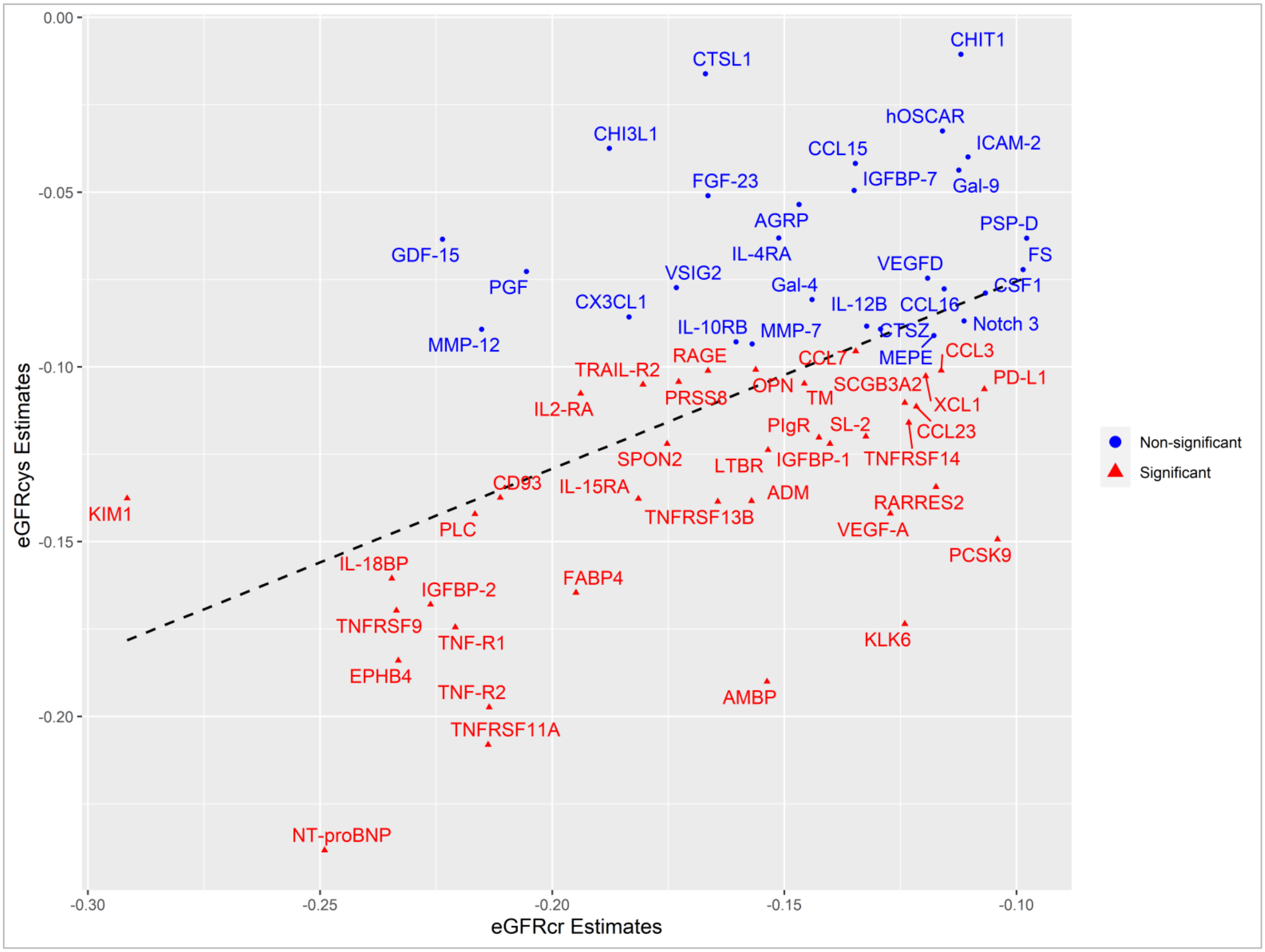

Figure S7. Association of 66 proteomic biomarkers with eGFRcr-based CKD incidence. Only 66 biomarkers significantly associated with the annual rate of change in eGFRcr were used to investigate their associations with incident CKD. The biomarkers are sorted by the magnitude of HRs.

Abbreviations: CI, confidence interval; CKD, chronic kidney disease; eGFRcr, creatinine-based estimated glomerular filtration rate; HR, hazard ratio; FDR, Benjamini–Hochberg false-discovery rate. Full names of the biomarkers can be found in Additional file 1: Table S1.

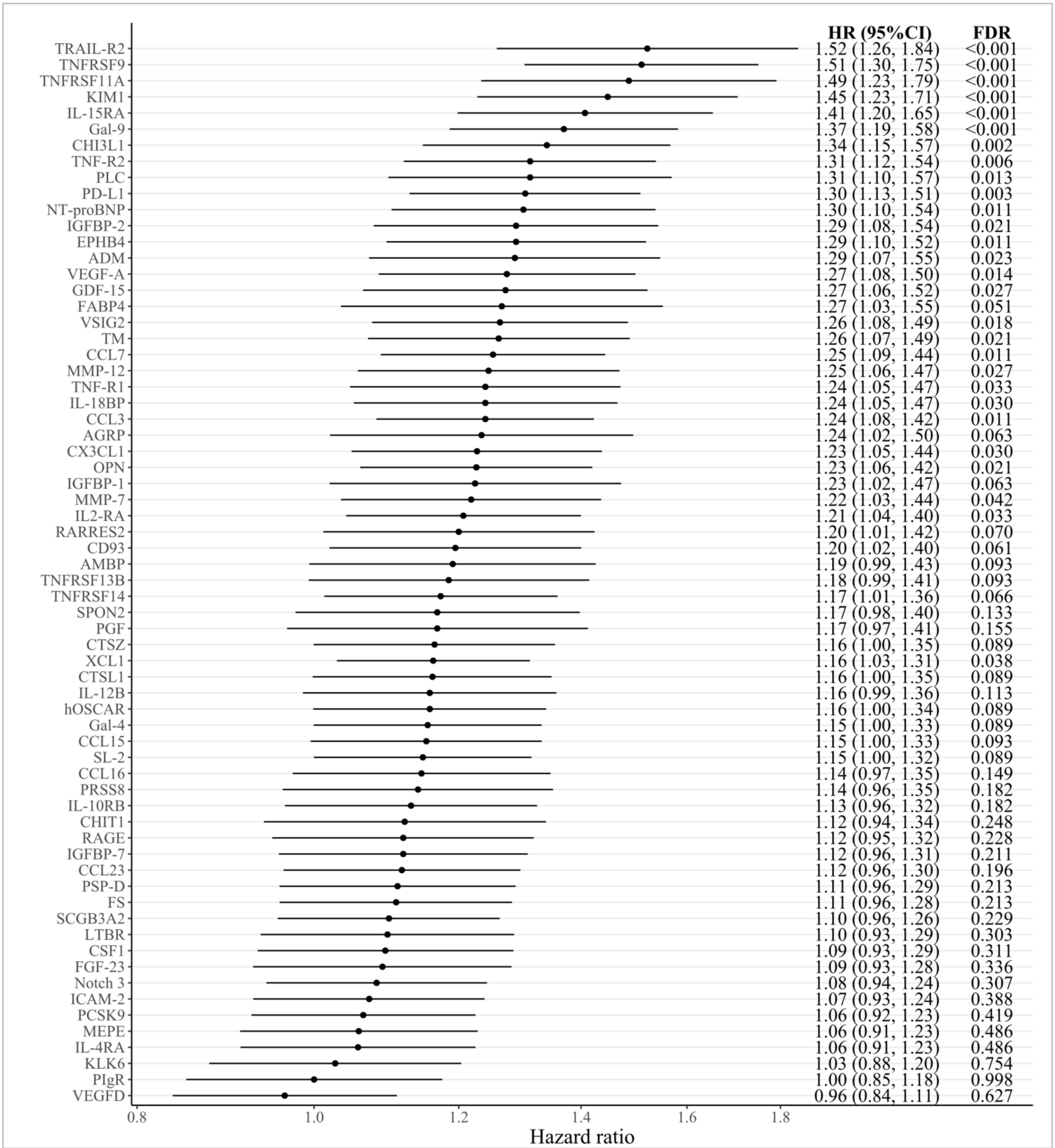

Figure S8. Pairwise correlation matrix between the 21 identified proteomic biomarkers. Correlation between the 21 biomarkers associated with the annual rate of change in eGFRcr, annual rate of change in eGFRcys, and incident CKD (Figure 3).

Abbreviations: CKD, chronic kidney disease; eGFRcr, creatinine-based estimated glomerular filtration rate; eGFRcys, cystatin C-based estimated glomerular filtration rate. Full names of the biomarkers can be found in Additional file 1: Table S1.

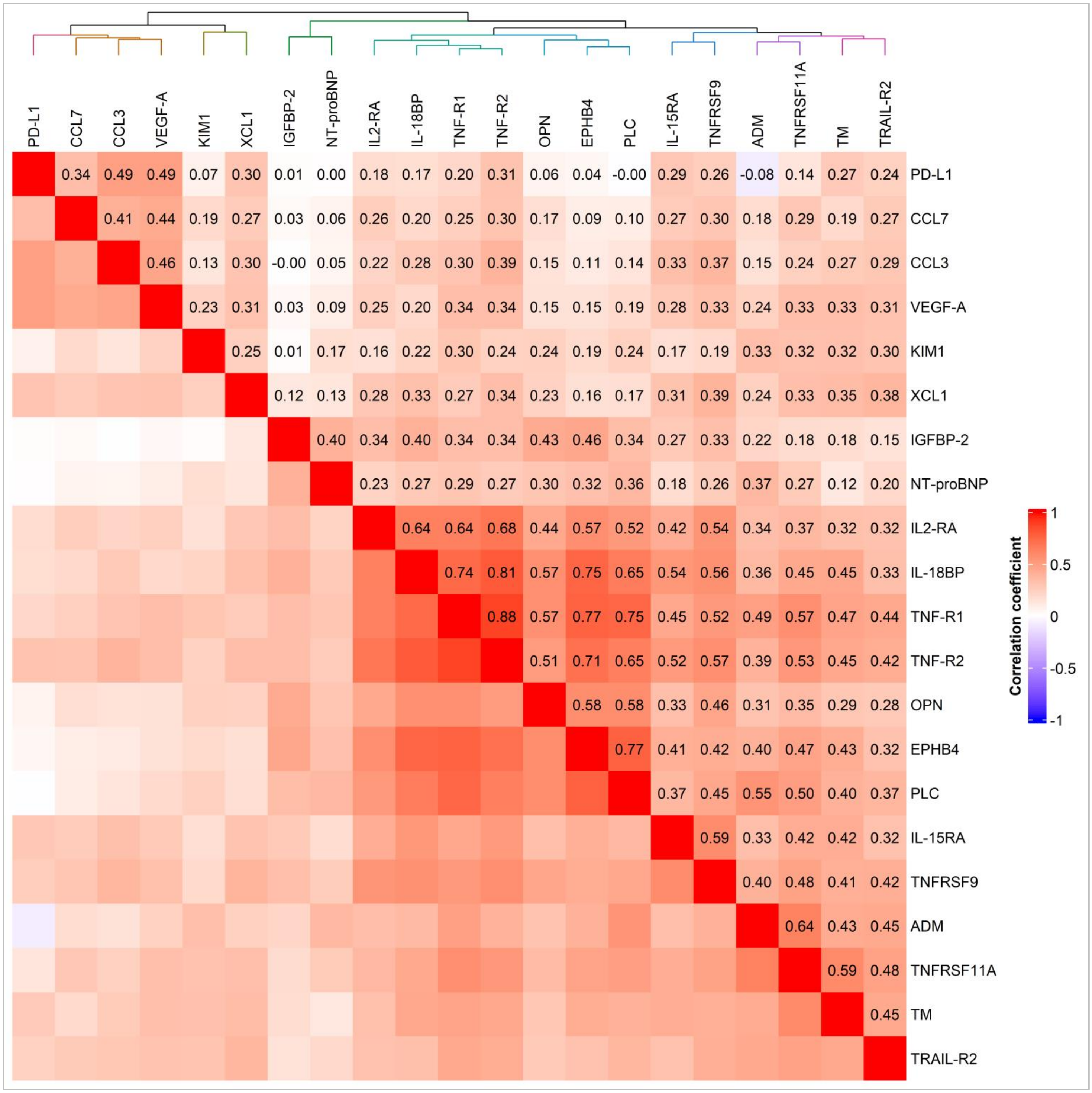

Figure S9. Pathway enrichment analysis of the 21 identified biomarkers showing top biological processes related to kidney function. The 21 biomarkers significantly associated with the annual rate of change in eGFRcr, annual rate of change in eGFRcys, and incident CKD (Figure 3), were included in the pathway enrichment analysis. The y-axis signifies the top 15 biological processes in kidney function. The x-axis is the -log10 of the FDR.

Abbreviations: CKD, chronic kidney disease; eGFRcr, creatinine-based estimated glomerular filtration rate; eGFRcys, cystatin C-based estimated glomerular filtration rate; FDR, Benjamini–Hochberg false-discovery rate.

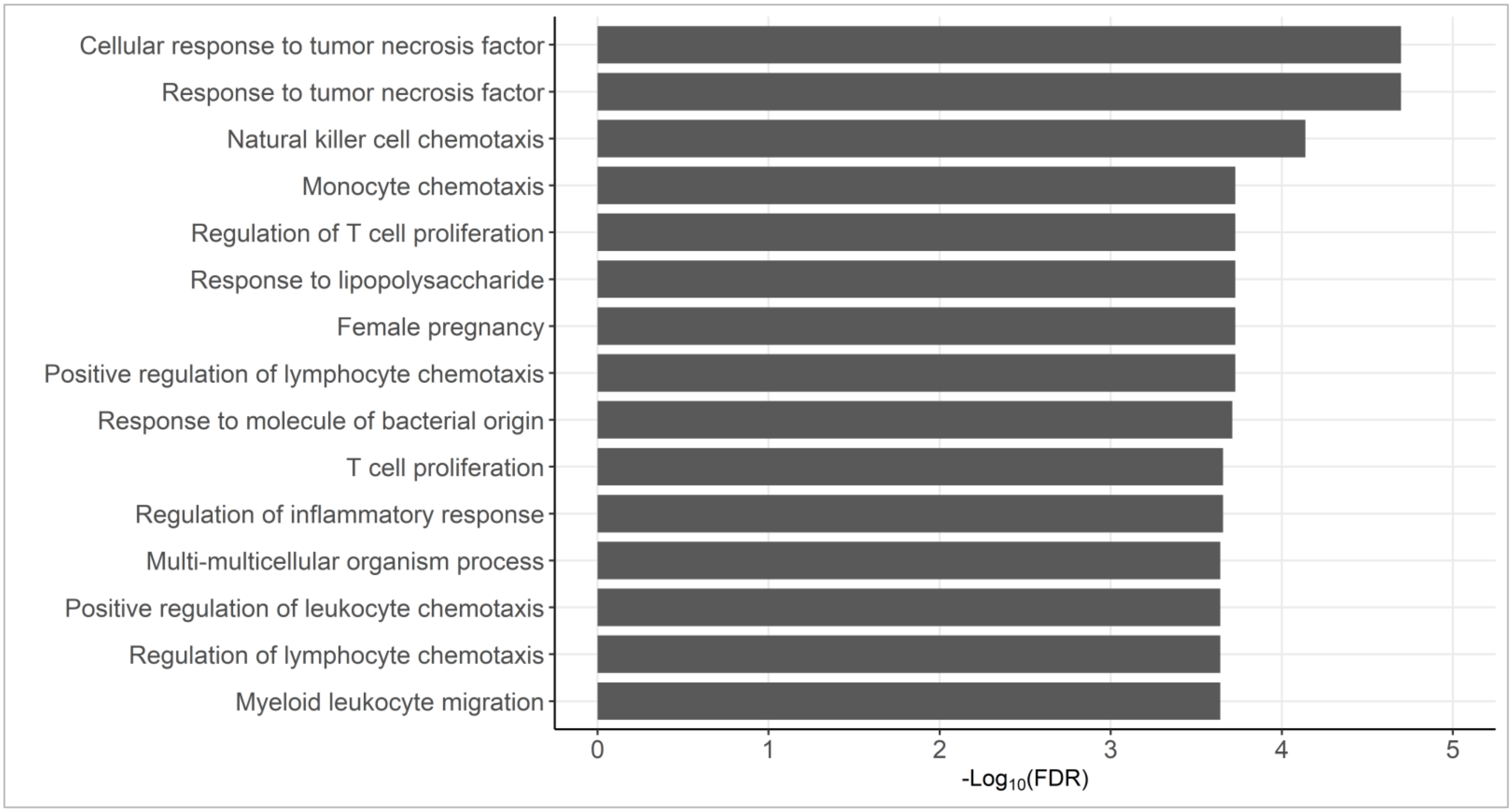

Supplement: Supplementary file 2 — Additional file 2: Text S1. Assessment of kidneyoutcomes; TextS2. Inverse probabilityweighting; Text S3. Mendelianrandomization analysis; Figure S1. Exampleof the annual rate of change in eGFR for each participant; Figure S2. Flowchart of statistical analyses; Figure S3. Genetic instrument selection and data harmonization forMendelian randomization analysis; FigureS4. Distribution and correlation between the annual rate of change ineGFRcr and eGFRcys; Figure S5. Overlapof proteomic biomarkers between biomarkers associated with the annual rate ofchange in eGFRcr in several sensitivity analyses; Figure S6. Longitudinal associations between 66 proteomicbiomarkers and the annual rate of change in eGFRcys; Figure S7. Association of 66 proteomic biomarkers with eGFRcr-basedCKD incidence; Figure S8. Pairwisecorrelation matrix between the 21 identified proteomic biomarkers; Figure S9. Pathway enrichment analysisof the 21 identified biomarkers showing top biological processes related tokidney function. [file 12916_2023_2962_MOESM2_ESM.pdf]
